# Lanthanide (Ce, Nd, Eu) environments and leaching behavior in borosilicate glasses

**DOI:** 10.1038/s41598-021-92777-w

**Published:** 2021-06-24

**Authors:** M. Fabian, F. Pinakidou, I. Tolnai, O. Czompoly, J. Osan

**Affiliations:** 1 Centre for Energy Research, Konkoly Thege St. 29-33, Budapest, 1121 Hungary; 2 School of Physics, Aristotle University of Thessaloniki, 54124 Thessaloniki, Greece

**Keywords:** Structural materials, Glasses

## Abstract

Borosilicate glasses will be used to stabilize the high-level radioactive wastes for disposal in a geological repository. Understanding the effects of actinide addition to a borosilicate glass matrix is of great importance in view of waste immobilization. Lanthanides were considered as chemical surrogates for actinides. The local structures of Ce^3+^, Nd^3+^ and Eu^3+^ ions in borosilicate glass, have been investigated by synchrotron radiation based techniques. The atomic parameters, such as bond lengths and coordination environments derived from X-ray diffraction, in combined with Reverse Monte Carlo simulations show correlation with X-ray absorption fine structure data. The lanthanide ions are in the common network with the tetrahedral SiO_4_ and with the mixed trigonal BO_3_ and tetrahedral BO_4_ units. Second neighbor atomic pair correlations reveal that the Ce^3+^, Nd^3+^ and Eu^3+^ ions are accommodated in both Si and B sites, supporting that the lanthanide-ions are stabilized in the glass-matrix network. Microscopy and microanalysis provided information on the amorphous state and on the major elemental composition of the high lanthanide-concentration samples. The release of matrix components (Si, B, Na, Ba, Zr) is higher than that of lanthanides (Ce, Nd, Eu). Both types of elements show a decreasing release tendency with time.

## Introduction

Reprocessing of spent nuclear fuel (SNF) generates high-level radioactive wastes (HLW), mainly actinides in different chemical qualities and quantities. HLW must be stabilized and isolated from the biosphere in very durable and stable solid matrix. After reprocessing of SNF, U and Pu are converted into a Mixed Oxide material, used as nuclear fuel and the remaining long-lived radioactive actinide elements (such as Th, U, Np, Pu, Am and Cm) must be treated^1^. Borosilicate glasses, thanks to their mechanical-chemical durability, safety and economical preparation way, are widely accepted as candidates for immobilization of HLW materials^2–4^. During the investigations we need to understand the incorporation way of radioactive chemical constituents, the chemical durability and the capacity of the matrix to retain radionuclides. Borosilicate glasses remain the best waste forms for the immobilization of radionuclides, they exhibit excellent mechanical and structural properties, but their chemical durability was not improved.

Lanthanide rare-earth elements (Ln: Ce^3+^, Nd^3+^ and Eu^3+^) are used as minor actinide surrogates, since they exhibit very similar chemical properties^5,6^. Ce^3+^ can be used to model Pu^3+^, while Nd^3+^ to model Cm^3+^, and Eu^3+^ to model Am^3+^, because of their comparable ionic radii^7^. It is characteristic that, Ce is easier to reduce to its trivalent state at higher preparation temperatures, leading to the presence of both Ce^3+^ and Ce^4+^ ions in the final glass^6^. In this paper, structural and microstructural results concerning the incorporation of Ce^3+/4+^, Nd^3+^ and Eu^3+^ ions in borosilicate glasses and the local environment of Ln ions are presented.

The 90/70wt%[55SiO_2_·10B_2_O_3_·25Na_2_O·5BaO·5ZrO_2_(mol%)]·10/30wt% Ln-oxide (CeO_2_, Nd_2_O_3_, Eu_2_O_3_) (denoted as follows: *Matrix-Ce10*, *Matrix-Nd10*, *Matrix-Eu10* (for 90wt% Matrix-glass + 10wt% of the respective Ln oxide), and *Matrix-Ce30*, *Matrix-Nd30*, *Matrix-Eu30* (for 70wt% Matrix-glass + 30wt% of the respective Ln oxide) was earlier synthesized and its basic structural properties were presented^8,9^. A comprehensive work on the Ln-glass systems was presented in Ref^9^, focusing on the atomic parameters of the host glassy system and the effect/influence of lanthanides on the basic network structure of the glass matrix upon incorporation of Ce, Nd and Eu ions.

The present work is a follow-up article of Ref^9^ where the local environment around three lanthanides (Ce^3+^, Nd^3+^ and Eu^3+^) occurring in matrix glass is studied. Several experiments were performed: X-ray diffraction measurements (XRD) combined with Reverse Monte Carlo (RMC) simulations, X-ray absorption fine structure (XAFS), Scanning electron microscopy (SEM) and energy-dispersive X-ray spectroscopy (EDX) analysis to determine lanthanide ion environments, their incorporation and stability behavior into the glassy matrix with the objective to model the incorporations and behaviors of actinides on the glass matrix. The chemical durability of the borosilicate glasses containing Ce^3+^, Nd^3+^ and Eu^3+^ was studied using ASTM C1285-02 product consistency test to study their elemental release characteristics under controlled conditions.

## Results and discussion

### SEM/EDX analysis

**Figure 1:**
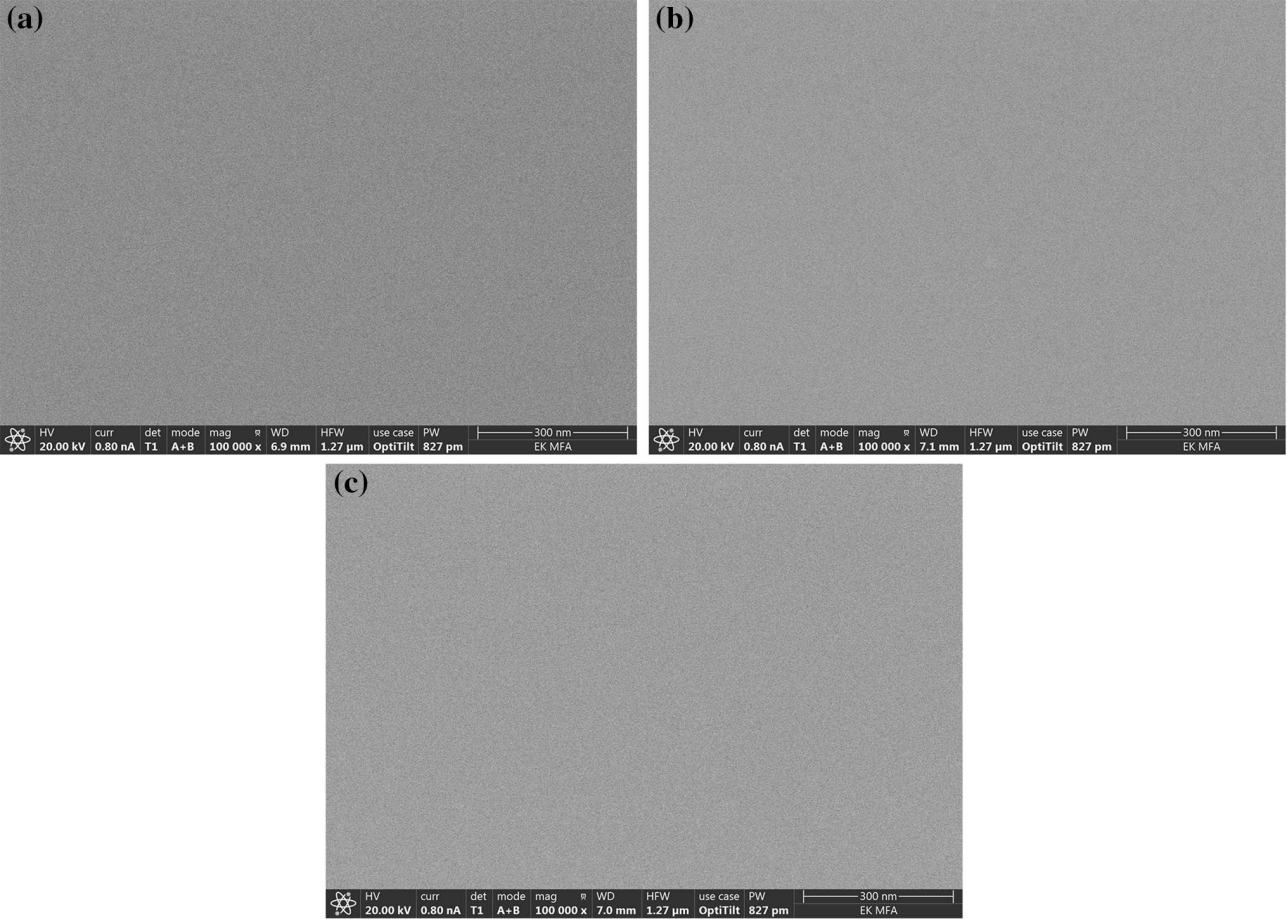
SEM backscattered electron images (atomic number contrast) at a 100,000 × magnification on the surface of the Matrix-Ce30 (**a**), Matrix-Nd30 (**b**) and Matrix-Eu30 (**c**) samples.

**Figure 2:**
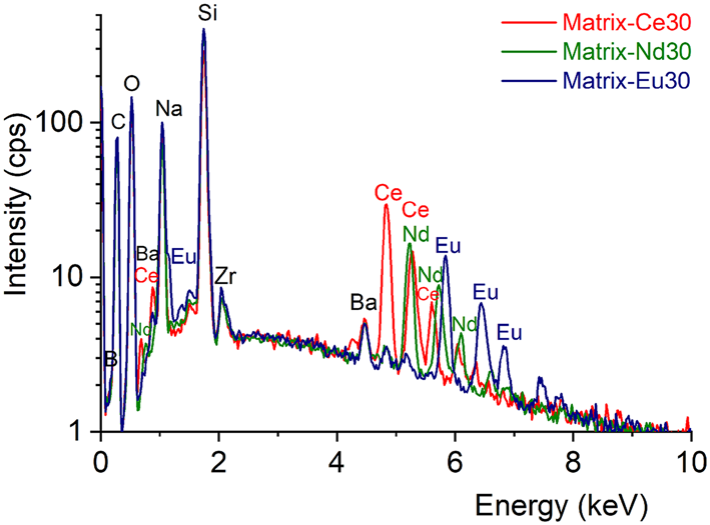
EDX spectra of the Matrix-Ce30 (blue), Matrix-Nd30 (yellow) and Matrix-Eu30 (red) samples.

Crystallization and homogeneity of the glasses were investigated by applying SEM analysis. SEM analyses were conducted on the Matrix-Ln30 samples for the microstructural investigation of the crystallization behavior of the glassy system, surface SEM micrographs are shown in Fig. 1. The surface SEM micrograph of each bulk samples shows typical amorphous nature, a homogeneous texture. Results indicate that no crystallization or phase separation was detected, which is a promising consequence for the application envisaged here. Using EDX it was possible to verify the composition of the Matrix-Ln30 glasses formed in the bulk. EDX spectra show that the intensities of lanthanide *L* X-ray lines are consistent with their content (*cf.* Fig. 2).

### Lanthanide environments with XRD and Reverse Monte Carlo simulations

The resulting Ln-containing glasses are proved to be transparent and fully amorphous, no crystalline phase can be observed supporting that the synthesis by employing melt quench technique is suitable. Detailed sample preparation steps are described in our previous work^9^. A comparison of RMC fitted total structure factors with experimental XRD data are performed. The structure factors of Matrix-Ln10 and Matrix-Ln30 glasses, within the series, do not differ from each other. These similarities come from the very close weighting factors, *w*_ij_ (defined in Eq. (1)) of the partial structure factors, *S*_ij_(*Q*) (defined in Eq. (2)):

1 $$S(Q) = \sum\limits_{{i,j}}^{k} {w_{{ij}} } S_{{ij}} (Q)$$

2 $$w_{{ij}} ^{{XRD}} (Q) = \frac{{c_{i} c_{j} f_{i} (Q)f_{j} (Q)}}{{\left[ {\sum\limits_{{i,j}}^{k} {c_{i} f_{j} (Q)} } \right]^{2} }}$$

where *c*_*i*_*, c*_*j*_, *f*_*i*_*(Q), f*_*j*_*(Q)* and *k* are the molar fractions, the X-ray scattering amplitudes, and the number of elements in the sample, respectively, and *Q* is the momentum transfer. The X-ray scattering amplitudes are *Q*-dependent^10^. Table 1 contains the input parameters, the weighting factors for the most important atomic pairs, *w*_ij_ used in the RMC run for the glass samples at *Q* = 0.86 Å^−1^. The Si–O, Ce–O, Nd–O, Eu–O and O–O atomic pairs and the Si–Ce, Si–Nd, Si–Eu second neighbor distances contribute significantly to the XRD intensity providing thus, accurate information on the lanthanide environments.

**Table 1:** XRD weighting factors, *w*_ij_(%) at *Q* = 0.86 Å^-1^.

Samples	Weighting factor, *w*_*ij*_ (%)					
	Si–O	B–O	Ce–O	Si–Ce	B–Ce	O–O
Matrix-Ce10	16.49	2.12	7.40	3.61	0.46	16.82
Matrix-Ce30	12.26	1.54	16.50	7.21	0.91	14.04

	Si–O	B–O	Nd–O	Si–Nd	B–Nd	O–O
Matrix-Nd10	16.29	2.06	7.75	3.85	0.48	16.40
Matrix-Nd30	11.78	1.42	16.86	7.64	0.96	12.99

	Si–O	B-O	Eu–O	Si–Eu	B–Eu	O–O
Matrix-Eu10	16.26	2.08	7.84	3.90	0.49	16.36
Matrix-Eu30	11.70	1.48	16.95	7.73	0.97	12.90

For the RMC starting model a disordered atomic configuration was built up with a simulation box containing 10,000 atoms using experimentally obtained density data (*ρ*_0_). The measured densities were 0.065 atoms Å^−3^ (half-box value: 27.59 Å) for Matrix-Ce10, 0.068 atoms Å^−3^ (half-box value: 26.26 Å) for Matrix-Nd10, 0.072 atoms Å^−3^ (half-box value: 25.89 Å) for Matrix-Eu10, 0.069 atoms Å^−3^ (half-box value: 26.79 Å) for Matrix-Ce30, 0.071 atoms Å^−3^ (half-box value: 26.01 Å) for Matrix-Nd30 and 0.078 atoms Å^−3^ (half-box value: 25.21 Å) for Matrix-Eu30. In the course of the RMC fitting, one constraint was chosen for minimum atom–atom distances to avoid physically invalid overlapping. Characteristic values obtained in our previous works were used as initial cut-off distances for the RMC simulation procedure. These distances were reported for binary 70SiO_2_–30Na_2_O^11^ and 75B_2_O_3_–25Na_2_O glasses^12^, for the 45SiO_2_–25Na_2_O–10B_2_O_3_–5BaO–5ZrO_2_ glass^8^ and for the neutron diffraction data of the present series^9^.

For each sample about 25 RMC simulation steps were generated with close to 1,300,000 accepted moves of each atom.

**Figure 3:**
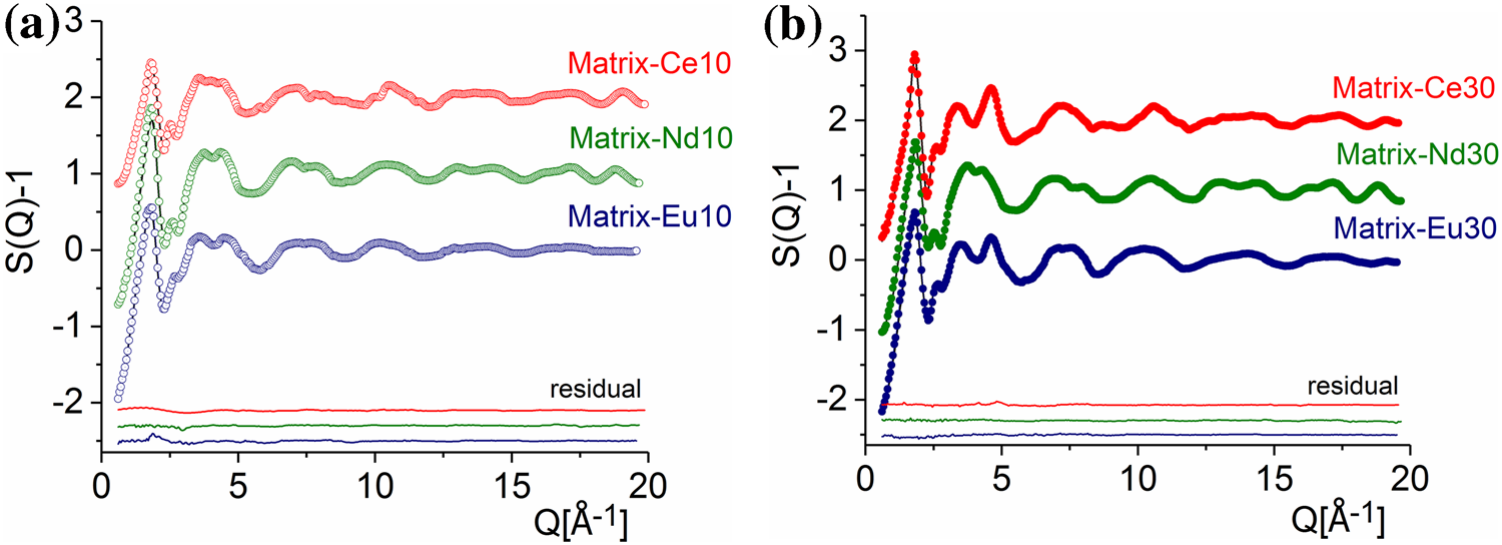
Total structure factors derived from XRD diffraction on Matrix-Ce10 (red circle), Matrix-Nd10 (green circle), Matrix-Eu10 (blue circle) (**a**) and Matrix-Ce30 (red dot), Matrix-Nd30 (green dot), Matrix-Eu30 (blue dot) (**b**) glasses (colour symbols) and RMC fits (black solid lines). For better visibility, the curves were shifted vertically.

The structure factors calculated by the RMC technique (black line) provided an excellent fit of the experimental XRD ones (colour symbols) (Fig. 3). The shape and features of the structure factors show similarities within the Ln-series.

From the RMC simulation several first and second neighbor partial atomic pair-correlation functions, *g*_*ij*_*(r)* and coordination number distributions, *CN*_*ij*_ have been revealed with a fairly good stability and statistics.

The atomic partial pair-correlation distances are summarized in Table 2. Structural data for the Matrix-glass sample^8^ and the neutron diffraction data for the present series were reported earlier^9^.

**Table 2:** Interatomic distances, *r*_ij_(Å) obtained from RMC simulations. The errors are estimated from the reproducibility of multiple RMC runs on each sample.

Samples	Interatomic distance, *r*_ij_(Å)					
	**Si–O**	**B–O**	**Ce–O**	**Si–Ce**	**B–Ce**	**O–O**
**Matrix-Ce10**	1.60 ± 0.01	1.35/1.7 ± 0.05	2.45/3.05 ± 0.05	3.45/4.05 ± 0.05	2.60/3.05 ± 0.1	2.40 ± 0.05
**Matrix-Ce30**	1.61 ± 0.01	1.35/1.75 ± 0.05	2.45/3.05 ± 0.05	3.50/4.10 ± 0.05	2.65/3.0 ± 0.1	2.25 ± 0.05

	**Si–O**	**B–O**	**Nd–O**	**Si–Nd**	**B–Nd**	**O–O**
**Matrix-Nd10**	1.60 ± 0.01	1.35/1.65 ± 0.05	2.35 ± 0.05	2.80/3.45 ± 0.05	2.65/3.15 ± 0.1	2.35 ± 0.05
**Matrix-Nd30**	1.60 ± 0.01	1.37/1.70 ± 0.05	2.35 ± 0.05	2.80/3.25 ± 0.05	2.65/3.15 ± 0.1	2.35 ± 0.05

	**Si–O**	**B–O**	**Eu–O**	**Si–Eu**	**B–Eu**	**O–O**
**Matrix-Eu10**	1.60 ± 0.01	1.35/1.70 ± 0.05	2.33 ± 0.05	3.25/3.75 ± 0.05	3.05 ± 0.1	2.35 ± 0.05
**Matrix-Eu30**	1.60 ± 0.01	1.35/1.65 ± 0.05	2.35 ± 0.05	3.25/3.70 ± 0.05	3.10 ± 0.1	2.35 ± 0.05

For Si–O a covalent bond length at 1.60 ± 0.01 Å was revealed for all studied glasses, showing an excellent agreement within the uncertainties and consistent with data reported in the literature^9, 12–14^. From the RMC analysis it was found that Si atoms are tetrahedrally coordinated with oxygen atoms. The obtained coordination number is in the range of 3.89–4.02, i.e. 3.89 ± 0.02 (Matrix-Ce10), 3.95 ± 0.02 (Matrix-Nd10), 3.98 ± 0.02 (Matrix-Eu10), 3.97 ± 0.02 (Matrix-Ce30), 4.00 ± 0.02 (Matrix-Nd30), 4.02 ± 0.02 (Matrix-Eu30). The Si surroundings predict that the glass network is built up from very stable SiO_4_ units in all studied glasses. Increasing the Ln-ion concentration causes an increase in the proportion of Si^4+^ species, a tendency correspondent to the ND data previously reported^9^.

The B–O atomic pair correlation functions show characteristic double peaks at 1.35 ± 0.05 Å and at 1.65 ± 0.05 Å, with significantly different relative intensities for varying Ln concentrations. Moreover, earlier studies also concluded that B–O surroundings are influenced by changes of network modifier cation concentrations^15–18^. For B–O distributions in both threefold coordination (^[3]^B as BO_3_) and fourfold coordination (^[4]^B as BO_4_) with oxygen surroundings have been revealed. The B–O coordination numbers are in the range of 3.46–3.95, i.e. 3.46 ± 0.05 (Matrix-Ce10), 3.55 ± 0.05 (Matrix-Nd10), 3.71 ± 0.05 (Matrix-Eu10), 3.82 ± 0.05 (Matrix-Ce30), 3.86 ± 0.05 (Matrix-Nd30) and 3.95 ± 0.05 (Matrix-Eu30). The B–O speciation revealed that the borosilicate glass network contains trigonal BO_3_ and tetrahedral BO_4_ units, therefore it is expected that the ratio of BO_3_/BO_4_ plays an important role in the formation of the glassy network. Modifiers such as lanthanides^9,19,20^, initiate the structural transformation and establish the ability of the glass matrix to incorporate heavy ions (e.g. radionuclides). The changes in boron coordination numbers revealed that a structural transformation of BO_3_ to BO_4_ occurs in the glass network by an increase in CeO_2_, Nd_2_O_3_ or Eu_2_O_3_ concentration which supports our previous ND results^9^. Based on the RMC calculations the basic network structure was established as mixed ^[3]^Si–O–^[3]^B and ^[3]^Si–O–^[4]^B linkages^8,9,13,21^, which lie in excellent agreement with our neutron diffraction data, i.e. the network structure is stable for all studied samples.

One characteristic O–O correlation peak was identified at 2.35 ± 0.05 Å, which appears at the same distance for all compositions (*cf.* Table 2).

**Figure 4:**
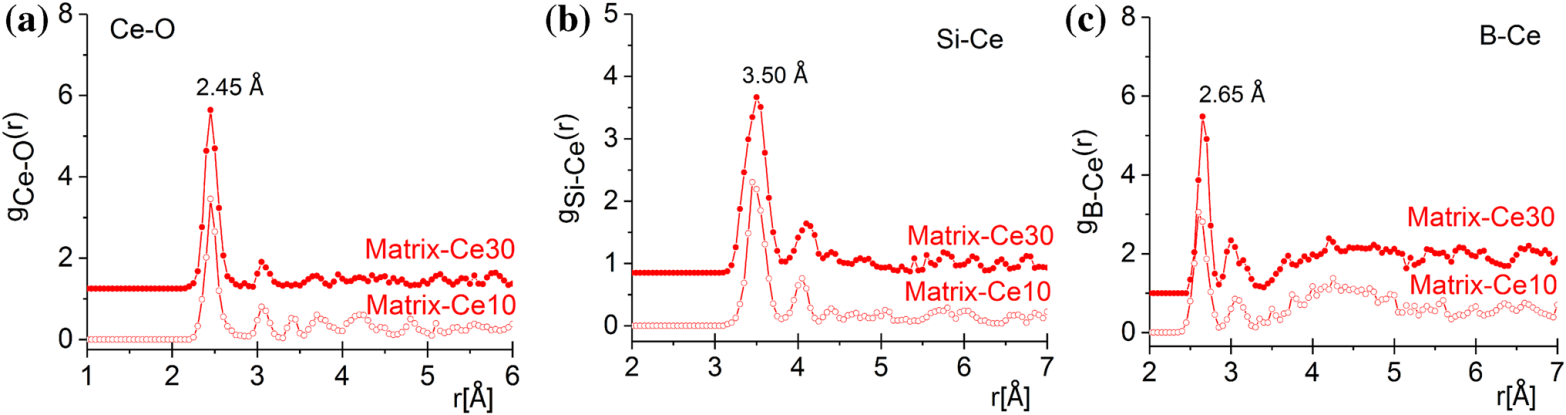
The partial atomic pair correlation functions obtained by RMC simulation for the Ce–O (**a**), Si-Ce (**b**) and B-Ce (**c**) for the Matrix-Ce(10/30) glasses. For better visibility, the curves were shifted vertically.

**Figure 5:**
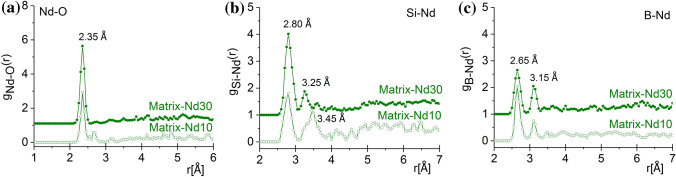
The partial atomic pair correlation functions obtained by RMC simulation for the Nd-O (**a**), Si-Nd (b) and B-Nd (c) for the Matrix-Nd(10/30) glasses. For better visibility, the curves were shifted vertically.

The lanthanide surroundings were obtained with a great stability from the RMC calculations, the Ln-environment was achieved with a good reproducibility due to their high weight in the XRD experiment. Figure 4a displays *g*_Ce-O_(*r*), where one well-resolved peak appears at 2.45 ± 0.05 Å, with a shoulder at 3.05 ± 0.05 Å. The Ce–O atomic distances indicate the presence of Ce^3+^–O, in agreement with previous results reporting Ce^3+^–O bond length of 2.48 Å^22^. The average Ce coordination is found equal to 6.05 ± 0.1 and 6.3 ± 0.1 for the samples Matrix-Ce10 and Matrix-Ce30 samples, respectively (*cf.* Fig. 7a); these values are close to the those reported for Ce^3+^–O average coordination numbers which range from 6.2 to 6.5^23^. Clear correlations can be found for Si-Ce at 3.45/4.05 ± 0.05 Å and for B-Ce at 3.50/4.10 ± 0.1 for Matrix-Ce10 and for Matrix-Ce30, respectively (lower than 3.70 Å reported in Ref^23^). This is an indication that the Ce atoms correlate with the network former Si and B atoms, as shown in Fig. 4b,c. The atomic pair correlation function of Nd-O indicates a first neighbor distance at 2.35 ± 0.05 Å, which is shorter than that previously reported values^22,24^ but longer than the distance determined from the ND measurements^9^ (see Fig. 5a). The average Nd-O coordination number is found equal to 7.3 ± 0.1 and 7.8 ± 0.1 for samples Matrix-Nd10 and Matrix-Nd30 samples, respectively (*cf.* Fig. 7b), which is higher than the respective obtained earlier from the ND data. The Si-Nd and B-Nd second neighbour distances were obtained at 2.80 ± 0.05 Å and 2.65/3.15 ± 0.1 Å, for the Matrix-Nd10 and for the Matrix-Nd30 concentrations, respectively (*cf.* Fig. 5b,c).

**Figure 6:**
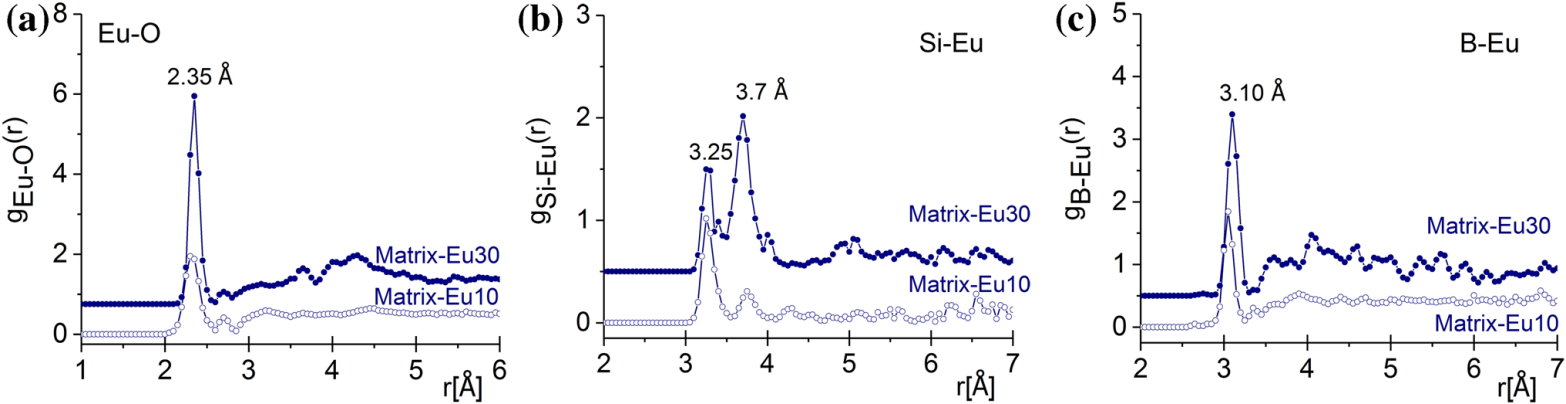
The partial atomic pair correlation functions obtained by RMC simulation for the Eu–O (a), Si-Eu (**b**) and B-Eu (**c**) for the Matrix-Eu(10/30) glasses. For better visibility, the curves were shifted vertically.

**Figure 7:**
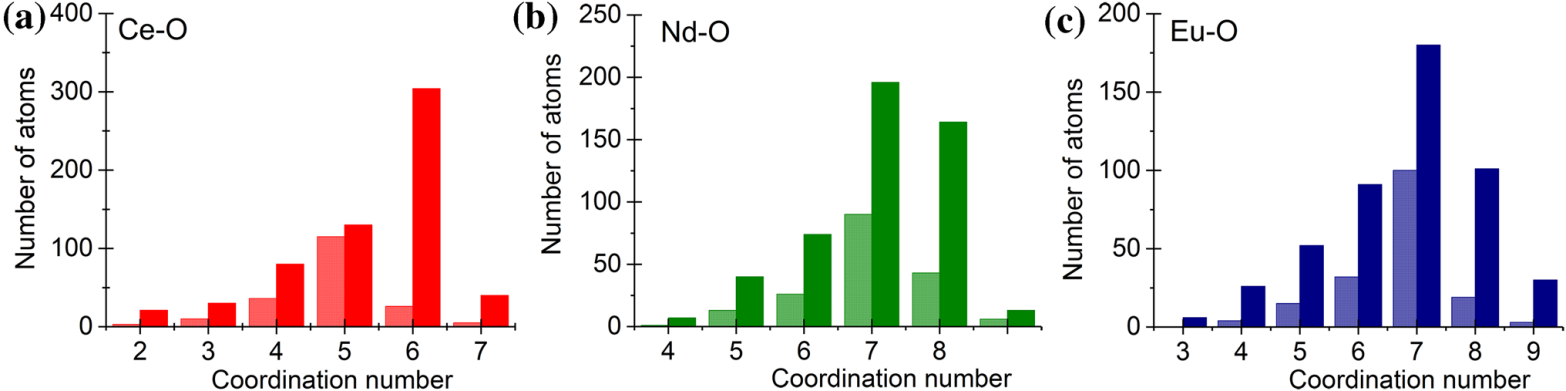
Ce–O (**a**), Nd-O (**b**) and Eu–O (**c**) coordination number distributions deduced from RMC modeling for the studied glasses: Matrix-Ce (red), Matrix-Nd (green) and Matrix-Eu (blue) glasses. Light colours: 10w%, dark colours: 30w% Ln-oxides.

Figure 6 presents *g*_Eu-O_(*r*) correlation function which shows one intensive peak at 2.33/2.35 ± 0.05 Å which is longer than the results obtained from ND data^9^. The peaks are above the value of 2.22 Å on the SiO_2_–Eu_2_O_3_ system deduced from a molecular dynamics simulation^25,26^. The Eu–O coordination numbers are equal to 7.2 ± 0.1 and 7.7 ± 0.1 for the Matrix-Eu10 and Matrix-Eu30 compositions, respectively (*cf.* Fig. 7c). Characteristic correlation functions were obtained between Si-Eu at 3.25/3.75 ± 0.05 Å and for B-Eu at 3.05/3.10 ± 0.1 Å for the Matrix-Eu10 and for the Matrix-Eu30 concentrations, respectively (*cf.* Fig. 6b,c).

Thanks to the relatively high XRD weighting factor of the Ln based connections, i.e. Ln–O and Si/B-Ln, well defined distances and stable glassy configuration can be revealed. The analysis of the second neighbour distances reveal that the B-Ln distances are shorter than the Si-Ln connections, suggesting that the Ln-ions preferentially connect to a B atom through an oxygen atom. Both types of connections suggest that the Ce, Nd and Eu atoms are incorporated into the basic borosilicate glass structure, and they are bound to oxygen atoms at a relatively short distances.

### Oxidation state of lanthanides by XANES

**Figure 8:**
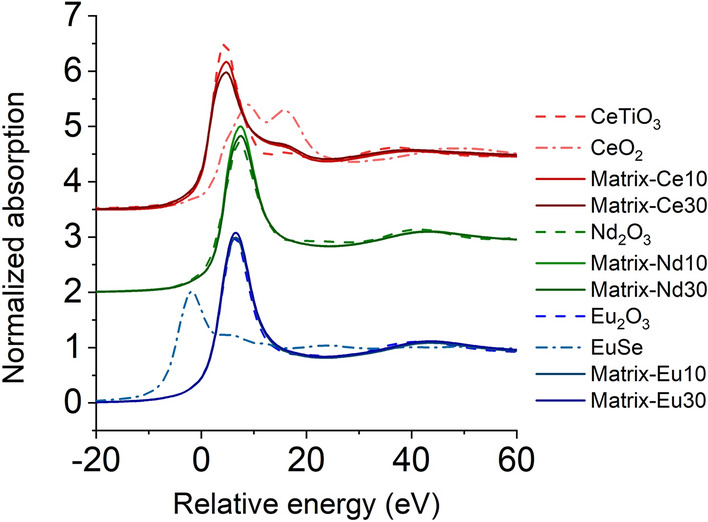
Comparison of Ce, Nd, and Eu L_III_ normalized XANES spectra of borosilicate glass samples (solid lines) and model compounds (dashed lines). The energy scale is relative to the respective L_III_ absorption edge (5723 eV for Ce, 6208 eV for Nd and 6977 eV for Eu).

Lanthanides were added to the borosilicate glass matrix components as oxides prior to preparation by melt-quenching, raw materials were melted at 1300 °C (Matrix-Eu10, Eu30) and 1450 °C (Matrix-Ce10, Ce30, Nd10, Nd30). Ce was initially added as Ce^4+^ (CeO_2_), which can be easily reduced to Ce^3+^ in the final glass structure, but a significant fraction can remain in the Ce^4+^ form^6^. For this reason, the oxidation state was investigated by XANES measurements at the Ce-*L*_III_ edge and least-squares fitting (LCF) with model compounds of known oxidation states, i.e. CeO_2_ for Ce^4+^ and CeTiO_3_ for Ce^3+^. XANES spectra of Ln-containing borosilicate glass samples and model compounds are presented in Fig. 8. LCF analysis revealed that 76% to 84% of Ce is present as Ce^3+^ in the glass and the amount of remaining Ce^4+^ ions is higher (24%) in the sample prepared with the addition of a higher concentration of CeO_2_ (Table 3). These findings are in accordance with results published for Ce-containing borosilicate glasses. Lopez et al.^5^ presented a dependence of the Ce oxidation state on the preparation temperature. 50% to 90% of Ce was found as Ce^3+^ for 1100 to 1400 °C melting temperatures when the glass was prepared in air atmosphere with 5% CeO_2_ added. The increase of the remaining Ce^4+^ fraction was also observed recently by Zhu et al.^27^ 53% to 69% Ce remained in the Ce^4+^ form for 5 to 20% CeO_2_ initially added. One should note that Zhu et al. used a considerably lower preparation temperature (1250 °C) than in the present work (1400 °C) explaining the difference in the obtained Ce^4+^/Ce_total_ molar ratios. Nd is present in the trivalent form in the glass samples as it is expected. The Eu-*L*_III_ XANES spectra of Matrix-Eu10, Eu30 are also similar to that of the initially added Eu_2_O_3_, although Eu is also redox-sensitive (*cf.* Fig. 8). LCF analysis using model compounds Eu_2_O_3_ and EuSe for Eu^3+^ and Eu^2+^, respectively, revealed that less than 1% of Eu is present as Eu^2+^ in the studied borosilicate glass samples. By comparison, Cicconi et al*.*^28^ investigated the Eu oxidation state in synthetic Eu-doped granitic and basaltic silicate glasses using XANES. The Eu^2+^/Eu_total_ molar ratios in glasses prepared in air at 1250–1650 °C were found to be in the range of < 1% to 22% strongly dependent on the composition.

**Table 3:** LCF results for Ce L_III_-edge XANES of borosilicate glass samples, using reference spectra of known oxidation states.

Sample	Ce^3+^ fraction	Ce^4+^ fraction
Matrix-Ce10	0.84 ± 0.01	0.16 ± 0.02
Matrix-Ce30	0.76 ± 0.02	0.24 ± 0.02

### Lanthanide environments by EXAFS

The EXAFS region of the energy scans at the Ce-*L*_III_ and Ce-*L*_II_ edges was highly affected by oscillations near the Ba-*L*_I_ absorption edge due to the high barium content of the borosilicate samples. Thus, in the case of the Ce-*L*_III_ spectra the available *k*-range is very limited, i.e. up to 7.5 Å^−1^, yielding very poor Fourier Transforms (FT) and unambiguous fitting results. For this reason, we focused on the Nd- and Eu-samples.

**Figure 9:**
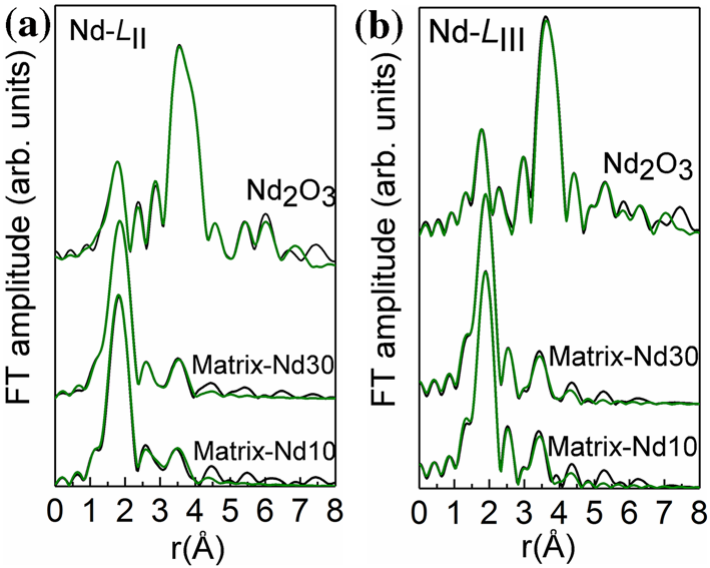
The Fourier transform spectra for Matrix-Nd10 and Matrix-Nd30 samples, at *L*_III_-edge (**a**) and at *L*_II_-edge (**b**). The green lines are the experimental data, and the black are the theoretical fits.

In the case of Nd-*L*_II_ and Nd-*L*_III_ edges, the fitting was performed simultaneously for the two glasses Matrix-Nd10 and Matrix-Nd30 (*cf.* Fig. 9a,b). The FT of the k^3^ × χ(k) Nd-*L*_III_- and Nd-*L*_II_- EXAFS spectra (*k*-range 3.0 to 9.5 Å^−1^ and 3.0 to 10.5 Å^−1^, respectively) of the studied glassy samples are shown in Fig. 9a,b, respectively. The spectra were fitted assuming a mixed bonding environment of Nd in the borosilicate glasses: (a) a fraction *x* of Nd atoms is tenfold coordinated surrounded by BO_3_ trigonal and BO_4_ tetrahedral units (the crystalline structure of Nd(BO_2_)_3_ was used)^29^, (b) the rest *y* = 1–*x* is eightfold coordinated and link to SiO_4_ units^30^. The fitting parameters where the percentage of Nd in different sites and the distances of the nearest neighbor atoms; the Debye–Waller factors (*σ*^2^) were free to vary for sample Matrix-Nd10 and remain constrained for sample Matrix-Nd30 and the analysis results for both edges are shown in the Tables 4 and 5.

**Table 4:** Structural parameters (coordination number, *N*; bond length, *r*; Debye–Waller factor, *σ*^2^; fraction of Nd link to borate, *x* and fraction of Nd link to silicate, *y*) for glasses Matrix-Nd10 and Matrix-Nd30 glasses obtained from the analysis of the Nd *L*_III_-edge EXAFS spectra. The asterisk denotes values that were kept fixed in the fitting process.

Nd-*L*_III_-edge			
	*N*	*r* (Å)	*σ*^2^ (× 10^−3^ Å^2^)
**Matrix-Nd10**		*x* = 0.808 ± 0.080	
O	10	2.33	6.5
B	10	3.12	15.5
O	4	4.03	2.8
Nd	2	4.35	13.3
		*y* = 0.192 ± 0.080	
O	8	2.20	5.1
Si	6	3.50	7.3
O	3	4.03	2.8
Nd	5	4.35	13.3
**Matrix-Nd30**		*x* = 0.797 ± 0.040	
O	10	2.33	6.5*
B	10	3.11	15.5*
O	4	4.04	2.8*
Nd	2	4.35	13.3*
		*y* = 0.203 ± 0.040	
O	8	2.20	5.1*
Si	6	3.48	7.3*
O	3	4.03	2.8*
Nd	5	4.34	13.3*
**Nd**_**2**_**O**_**3**_			
O	4*	2.33	9.4
O	4*	2.61	8.1
Nd	6*	3.74	6.1
Nd	6*	3.85	6.2
O	15	4.47	14.0
O	9*	5.05	14.05
Nd	6*	5.40	8.3
O	15*	5.93	8.0
Nd	18*	6.58	17.2
Nd	12*	7.18	13.3

**Table 5:** Structural parameters (coordination number, *N*; bond length, *r*; Debye–Waller factor, *σ*^2^; fraction of Nd link to borate, *x* and fraction of Nd link to silicate, *y*) for glasses Matrix-Nd10 and Matrix-Nd30 obtained from the analysis of the Nd *L*_II_-edge EXAFS spectra. The asterisk denotes values that were kept fixed in the fitting process.

Nd-*L*_II_-edge			
	***N***	***r* (Å)**	***σ***^**2**^** (× 10**^**‐3**^** Å**^**2**^**)**
**Matrix-Nd10**		*x* = 0.839 ± 0.080	
O	10	2.34	8.7
B	10	3.16	19.8
O	4	4.08	1.8
Nd	2	4.35	15.2
		*y* = 0.161 ± 0.080	
O	8	2.23	2.5
Si	6	3.45	2.4
O	3	4.08	1.8
Nd	5	4.35	15.2
**Matrix-Nd30**		*x* = 0.891 ± 0.060	
O	10	2.33	8.7*
B	10	3.16	19.8*
O	4	4.08	1.8*
Nd	2	4.35	15.2*
		*y* = 0.109 ± 0.060	
O	8	2.19	2.5*
Si	6	3.48	2.4*
O	3	4.08	1.8*
Nd	5	4.35	15.2*
**Nd**_**2**_**O**_**3**_			
O	4*	2.33	10.2
O	4*	2.61	9.4
Nd	6*	3.72	2.6
Nd	6*	3.85	3.5
O	15	4.44	18.3
O	9*	5.04	10.8
Nd	6*	5.39	6.5
O	15*	5.93	9.3
Nd	18*	6.58	16.9
Nd	12*	7.15	12.8

It is revealed that the majority of Nd^3+^ ions (~ 80 at%) is surrounded mainly by boron chains, a finding previously reported in alkali‐free borosilicate glasses at low Nd-concentrations^31^, while only a small fraction links to SiO_4_ silica units. This preference is not disturbed by the different chemical composition in the glasses. The environment of Nd is mainly borate (Nd–O and Nd–B at 2.33–2.35 Å and 3.16 Å, respectively); nevertheless, the Nd–O bond length is much shorter than the one reported in Ln-metaborate, Na-rich borate (2.44 and 2.48 Å) and Nd-doped borosilicate glasses^24^. To a much less extent, neodymium is present at a silicate site (Nd–O and Nd–Si distances equal to 2.18–2.23 Å and 2.45–3.48 Å, respectively); the Nd–O bond length is much shorter than 2.35 Å, a finding expected in a silicate yet alkali free glass, however Nd has a sixfold coordination^32^ or in natural garnets with Nd in trace amounts^33^. Finally, the invariance of the Nd–O distances in both glasses can be attributed to the same bonding environment of Nd, since a shorter bond length is expected when the Nd coordination number increases in glasses with varying Nd-concentration^34^.

**Figure 10:**
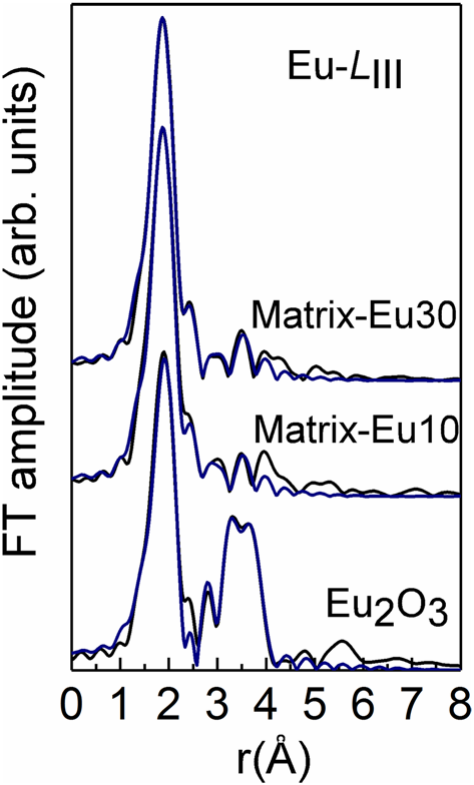
The Fourier transform spectra for Matrix-Eu10 and Matrix-Eu30 samples, at *L*_III_-edge. The blue lines are the experimental data, and the black are the theoretical fits.

As far as the Eu-*L*_III_ EXAFS spectra are concerned, the FT of the of the *k*^3^ × χ(*k*) Eu-*L*_III_-EXAFS spectra (*k*-range 3.0 to 11.5 Å^−1^) of the studied glassy samples are shown in Fig. 10. The fitting model used in this case, assumes a mixed bonding environment of Eu in the borosilicate glasses, (a) a fraction *x* of the Eu atoms is tenfold coordinated with O atoms and link to borate chains made up from [B_6_O_12_]_n_^6–^ structural units (the crystalline structure of LaB_3_O_6_, was used where Eu atoms substitute for La in the crystalline model)^29,35^ and (b) the rest *y* = 1–*x* form pentagonal bipyramids EuO_7_ that connect via corners to SiO_4_ units^36^. The fitting was again performed simultaneously for the two glasses Matrix-Eu10 and Matrix-Eu30 (*cf.* Fig. 10) and the fitting parameters where those reported previously for the Nd-*L*_II_ and Nd-*L*_III_ edges and the results are listed in the Table 6.

**Table 6:** Structural parameters (coordination number, *N*; bond length, *r*; Debye–Waller factor, *σ*^2^; fraction of Eu link to borate, *x* and fraction of Eu link to silicate, *y*) for glasses Matrix-Eu10 and Matrix-Eu30 obtained from the analysis of the Eu *L*_III_-edge EXAFS spectra. The asterisk denotes values that were kept fixed in the fitting process.

Eu-*L*_III_-edge			
	***N***	***R* (Å)**	***σ***^**2**^** (× 10**^**‐3**^** Å**^**2**^**)**
**Matrix-Eu10**		*x* = 0.466 ± 0.060	
O	10	2.37	14.2
B	10	3.15	14.5
O	4	4.00	2.6
Eu	2	4.03	15.1
		*y* = 0.534 ± 0.070	
O	7	2.31	6.3
Si	4	3.20	12.4
**Matrix-Eu30**		*x* = 0.532 ± 0.070	
O	10	2.35	14.2*
B	10	3.17	14.5*
O	4	4.00	2.6*
Eu	2	4.03	15.1*
		*y* = 0.468 ± 0.070	
O	7	2.30	6.3*
Si	4	3.20	12.4*
**Eu**_**2**_**O**_**3**_			
O	6*	2.37	10.8
Eu	6*	2.64	8.5
Eu	6*	3.82	13.0
O	8*	4.30	5.8

According to the EXAFS analysis results, the Eu^3+^ ions are equally distributed between the two different bonding geometries. Independently of the chemical composition, approximately half of the Eu ions form EuO_7_ bipyramids and preferentially link to SiO_4_ units of the silica matrix^37^; the same number of Eu is surrounded by 10 oxygen atoms, a fraction of which is commonly shared between Eu and B-centered tetrahedral and/or pyramids.

The Eu–O bond length in the two geometries lies in excellent agreement with the respective in the crystalline model used in the fitting process. Indeed, a shorter Eu–O distance is expected in the case of alkali silicate glasses doped with Eu^3+^ a^34,38^ compared to alkaline borate glasses. The EuO_7_ bipyramids share corners with four SiO_4_ silica units, located at a distance 3.20 Å. Additionally, BO_4_/BO_3_ units are found at the same distance from tenfold coordinated Eu^3+^ ions, i.e. at approximately 3.15–3.17 Å. Finally, no alkali metals are detected in the vicinity of rare‐earth sites, as it has been previously suggested for alkali silica glasses^34^.

In conclusion, EXAFS results at both Nd-*L*_II_,*L*_III_ and Eu-*L*_III_ edges deliver direct evidence that the Ln ions are not isolated from the silicate network. This is demonstrated by the absence of Ln atoms clustering in the glasses, with the presence of quasi-molecular complexes (molecular entities that modify the geometrical arrangement of the vitreous matrix to accommodate their own bonding requirements) containing Ln–O polyhedra with Ln–O–B and Ln–O–Si type of linkages. Finally, the difference in the ionic radii between Nd and Eu ions, accounts for the shorter Nd–O bond length compared to Eu–O in both environments.

Structural characterization of Ln-doped borosilicate glasses were performed by the combination of synchrotron and simulation techniques. The XAFS technique has been successfully applied, the structural factor being in good agreement with the ones obtained by RMC simulation. The local structure around Ln is nearly the same in both experiments. The partial pair correlation functions *g*_Nd-O_(*r*) and *g*_Eu-O_(*r*) shows similar peak positions, however the second neighbors from EXAFS strongly interfere with the XRD/RMC values. The differences are observed in the Nd and Eu average coordination numbers, which are lower in case of XRD data calculated by RMC simulation, as above 7, for each. Both experiments underline the Si- and B-environment around the Ln-atoms, which supports the incorporation of the Ln’s in the matrix network.

### Leaching tests

Concerning the release rates of surrogates in the studied glass waste forms, leaching tests in aqueous solutions were conducted on Matrix-glass and on Matrix-Ln30 sample series containing cerium, neodymium and europium as actinide surrogates for plutonium, americium and curium, based on ASTM C1285-02 protocol^39,40^. The results of the Product Consistency Tests (PCT), PCT-A and PCT-B on the glasses are shown in Table 7. In general, the stability of the surrogated structure is inversely proportional to the leaching rates, lower values represent a more durable glass waste form. Our data indicate that the releases of Ce, Nd and Eu are of a similar order of magnitude. The obtained release rates show a decreasing tendency in function of time, in all sample series similarly to those reported in the literature^41,42^. The normalized leaching rate of the Matrix-Nd30 sample at 7 days shows the same or lower magnitude compared to a Nd surrogated pyrochlore-borosilicate sample at 28 days^41^ and very similar rate values with calcium neodymium/cerium oxide silicate glass–ceramics^43^ and with the same range for glass–ceramic compositions with neodymium/cerium^44^. The decreasing tendency can be also identified for the Matrix-Ce30 sample test series and is in good agreement with the results reported in Ref^45^. Although our samples have substantially higher CeO_2_ content the order of magnitude of the normalized leaching rates are similar. Europium was used as a surrogate for curium and had the best results regarding the elemental release before the last sampling. On the tenth day the normalized leaching rates of Matrix-Nd30 and Matrix-Eu30 reached the same order of magnitude.

**Table 7:** Normalized elemental leaching rates in PCT-A and PCT-B tests from 3 to 10 days.

Sample	SA/V	NR_(Ln)_*	NR_(Si)_	NR_(B)_	NR_(Na)_	NR_(Ba)_	NR_(Zr)_
	(m^-1^)	(g/m^2^/d)					
**test A**							
Matrix-*7d*	1,96E + 03	–	5,43E−02	7,90E−02	3,18E−02	8,24E−04	5,39E−06
Matrix-Ce30-7d	1,73E + 03	2,66E−05	7,95E−01	6,06E−01	1,46E + 00	8,27E−03	4,31E−05
Matrix-Nd30-7d	1,65E + 03	2,78E−05	3,64E + 00	2,10E + 00	5,91E + 00	8,67E−02	4,53E−03
Matrix-Eu30-7d	1,61E + 03	6,49E−06	2,22E + 00	1,42E + 00	3,70E + 00	8,85E−02	4,98E−04
**test B**							
Matrix-3d	1,96E + 03	–	1,09E−01	1,61E−01	6,23E−02	1,60E−03	2,46E−05
Matrix-7d	2,94E + 03	–	3,59E−02	5,26E−02	2,24E−02	4,39E−04	5,99E−06
Matrix-10d	5,89E + 03	–	1,31E−02	2,01E−02	8,72E−03	2,18E−04	1,49E−06
Matrix-Ce30-3d	1,73E + 03	3,20E−05	1,29E + 00	1,37E + 00	3,41E + 00	4,90E−05	3,47E−05
Matrix-Ce30-7d	2,59E + 03	2,77E−05	7,69E−01	6,80E−01	1,62E + 00	5,60E−05	4,49E−05
Matrix-Ce30-10d	5,18E + 03	1,88E−05	3,18E−01	2,77E−01	6,44E−01	2,83E−05	2,35E−05
Matrix-Nd30-3d	1,65E + 03	1,48E−04	5,29E + 00	4,04E + 00	9,59E + 00	2,27E−03	9,77E−03
Matrix-Nd30-7d	2,47E + 03	2,48E−05	2,43E + 00	1,50E + 00	4,07E + 00	1,03E−03	3,98E−03
Matrix-Nd30-10d	4,95E + 03	6,04E−07	9,51E−01	5,37E−01	1,53E + 00	4,61E−04	1,19E−03
Matrix-Eu30-3d	1,61E + 03	7,07E−05	7,02E + 00	4,14E + 00	1,15E + 01	3,86E−03	2,08E−02
Matrix-Eu30-7d	2,42E + 03	7,93E−06	2,48E + 00	1,40E + 00	3,98E + 00	1,63E−03	4,73E−03
Matrix-Eu30-10d	4,84E + 03	1,98E−06	9,08E−01	5,00E−01	1,43E + 00	5,74E−04	1,35E−03

*Ce, Nd, Eu for the respective samples.

Matrix elements can be classified into two distinct groups based on the differences obtained in their release characteristics. The Si, B and Na elements tend to be more stable in the matrix without the presence of lanthanides and have lower normalized rates which is of great importance because these elements build up the basic glassy network. The PCT specification for borosilicate-based glasses is that the normalized release of Si, B and Na shall be < 2 g/m^2^/d using a PCT-A test^46^, our data are consistent with this recommendation, except Matrix-Nd30 sample. In case of borosilicates, for the high release elements like B and Na; and for the matrix element: Si, the reported values within the Ln-series are in the same order of magnitude. Boron is usually assumed to provide the best measure of the extent of glass reaction because of its high solubility. The release of boron occurred at an average rate of about 1.05 g/m^2^/d in case of PCT-A test, and about 1.22 g/m^2^/d for the PCT-B test. The boron and silicon solution concentrations increase with added Ln-oxide. PCT data on leaching of Si, B and Na from the Matrix-Ln30 glass series have similar leaching rates to those obtained in case of high-sodium content borosilicate glasses, where the leaching rates were 2.93E−02 g/m^2^/d, 4.05E−02 g/m^2^/d and 5.93E−02 g/m^2^/d for Si, B and Na, respectively^47,48^.

Our data presented in Table 7 are consistent with the data of Backhouse et al.^49^ received for the six-component alumino-borosilicate glass, where the dissolution rates were 1.31E−02 g/m^2^/d, 1.56E−02 g/m^2^/d and 1.45E−02 g/m^2^/d for Si, B and Na, respectively. Miekina^50^ studied leaching of B, Na and Si after 7 days for Ca- and Mg-borosilicate glasses. In that work, leaching values of 1.8–4.8 g/m^2^ and 0.3–0.4 g/m^2^ for B/Na and Si, respectively, which are lower than our results, however no lanthanides were added to those glasses. Leaching results obtained for SiO_2_–B_2_O_3_–Li_2_O–Na_2_O–ZnO glasses were presented by Vance et al.^51^ based on PCT-B and MCC-1 tests, where the normalized data were in the range of 0.5–25 g/m^2^/d for Si, 0.4–40 g/m^2^/day for B and 0.7–70 g/m^2^/d for Na, which are higher than our results. Based on the discussed values and recommended limits, our leaching results obtained for Si, B and Na elements, predict a stable glassy matrix in term of ASTM conditions.

The Zr and Ba elements act independently and show normalized release rate of similar order of magnitude in each sample. The normalized release of Zr is lower than that obtained for glass ceramic samples^44^, but for Ba we obtained the same numbers as calculated in the NEUP Report^44^. In case of Ba (test A/Matrix-Ce30) some discrepancy was found that is possibly connected to impurities.

PCT-A and PCT-B tests at the same time-range (7 days) show comparable values for Ln-elements and also for matrix-glass components.

## Conclusions

Borosilicate matrix glass (55SiO_2_·10B_2_O_3_·25Na_2_O·5BaO·5ZrO) with 10 wt% and 30 wt% addition of lanthanide oxides of CeO_2_, Nd_2_O_3_ and Eu_2_O_3_, respectively were investigated by synchrotron techniques (XRD, XAFS) and Reverse Monte Carlo simulation. The SEM investigation revealed the homogeneity and amorphous nature of the glasses. EDX analysis confirms the chemical compositions of the glasses, all of Ln-elements were identified. XANES results reveals that in addition to Nd and Eu which were originally added in the trivalent form, also Ce is mostly present in the reduced Ce^3+^ form with 16–24% of remaining Ce^4+^ content.

The RMC simulation of the experimental XRD data and XAFS analysis show that the Ln’s are stabilized in the silicate and borate network. The second neighbor connections between the network former Si/B-atoms and Ln-atoms supports the incorporation of the Ln’s in the basic network structure which are built up by mixed ^[3]^Si–O–^[3]^B and ^[3]^Si–O–^[4]^B linkages. The leaching characteristics of matrix-glass components and lanthanides are different but similar within the sample series. The leached amount of Ce, Nd and Eu decreases over time, supposing that the studied compositions at long-term can be a good choice for stabilization of surrogates of the selected (Pu, Am, Cm) actinides.

## Methods

### Scanning electron microscopy investigations

Powerful complementary characterization techniques capable of analyzing morphology and chemical composition at the surface and in the bulk of single nanoparticles are analytical electron microscopy, *i.e.* a combination of Scanning Electron Microscopy (SEM) with Energy Dispersive X-ray Spectroscopy (EDX). Morphology and shape information are derived from the detection of secondary electrons in SEM and analytical information from characteristic X-rays (EDX). Bulk composition is delivered by EDX with electron excitation because the excitation volume is typically at the micrometer scale. EDX can be used to obtain elemental information about the area of interest.

Microstructural characterization experiments were conducted with carbon coated bulk samples a Thermo Scientific Scios 2 DualBeam Sytem SEM with focused ion beam capabilities linked with an Oxford Instruments X-Max EDX attachment.

### X-ray diffraction experiments

The X-ray diffraction (XRD) measurements were performed on the P07 diffractometer at the PETRA III storage ring of Deutsches Elektronen-Synchrotron (Hamburg, Germany)^52^. The energy of the radiation was 47 keV (λ_0_ = 0.1263 Å). The raw data were corrected for detector dead time, background, absorption and variations in detector solid angle. The total structure factor, *S(Q)* was calculated by the PDFgetX3 software packages^53^.

### Reverse Monte Carlo simulations

The Reverse Monte Carlo (RMC) simulation is a powerful technique to build large 3D structural models in accordance with experimental data, in particular total structure factors (*S*(*Q*)) obtained from diffraction experiments. The *S(Q)* data were simulated for XRD diffraction by the RMC^2+^ code^54^. The RMC algorithm calculates the *g*_ij_(*r*) one-dimensional partial atomic pair correlation functions, and by inverse Fourier transformation, calculates the *S*_ij_(*Q*) partial structure factors as:

3 $$S_{{ij}} (Q) = 1 + \frac{{4\pi \rho _{0} }}{Q}\int\limits_{0}^{{r_{{\max }} }} {r\,\left[ {g_{{ij}} (r) - 1} \right]\,\sin Qr\;dr}$$

where *ρ*_0_ and *r*_max_ are the atomic number density and the half-edge-length of the simulation box in the RMC calculation. The actual computer configuration is modified by moving the atoms randomly until the calculated *S*(*Q*) and experimental data agree within the experimental error (*cf.* Eqs. (1) and (2)).

### X-ray absorption fine structure (XAFS) measurements

The measurements were performed at the XAFS beamline of Elettra synchrotron radiation facility (Trieste, Italy)^55^. Spectra covering both X-ray absorption near-edge structure (XANES) and extended X-ray absorption fine structure (EXAFS) regions were collected using a Si (111) monochromator around L_III_ and L_II_ absorption edges of Ce, Nd and Eu. The concentration of the elements of interest in the Ln-containing glass samples was sufficiently high for collection of XAFS spectra in transmission mode. Glass samples were investigated as 13-mm diameter pressed pellets with 50–100 mg/cm^2^ of lanthanide-containing glass and polyvinylpyrrolidone as a binder. Reference compounds CeO_2_, Ce_2_(SO_4_)_3_, Nd_2_O_3_ and Eu_2_O_3_ were measured in the form of pressed pellets as well.

All spectra were collected in air at room temperature. The Si(111) monochromator was tuned using energy steps of 5 eV below the absorption edge, 0.2 eV in the near-edge region and a *k*-constant step of 0.03 Å^−1^ in the extended region. The intensity of the incoming and transmitted X-rays were monitored by ionization chambers, using a 2 s dwell time per energy step. For each sample, multiple spectra have been collected and merged in order to increase the signal to noise ratio. The energy scale was calibrated by reference metal foils having K absorption edge within the energy regions of interest (Cr-*K* for Ce-*L*_III_,*L*_II_, Mn-*K* for Nd-*L*_III_,*L*_II_ and Fe-*K* for Eu-*L*_III_).

The oxidation state of cerium and europium in borosilicate glass samples was determined using linear combination fitting (LCF) of the XANES region based on reference spectra for model compounds of known oxidation states, CeO_2_ for Ce^4+^; Ce_2_(SO_4_)_3_ and CeTiO_3_ for Ce^3+^; Eu_2_O_3_ for Eu^3+^ and EuSe for Eu^2+^. For CeTiO_3_ and EuSe, published reference XANES spectra were used from Lytle database^56^ and XAS Data Library^57^, respectively. Background removal, normalization of XAFS spectra as well as LCF were performed using the Athena software package^58^. For EXAFS, FEFF8.2 was used to calculate the theoretical phase and amplitude functions for the scattering paths^59^, while curve fitting was carried out in both R- and k-space using FEFFIT^60^.

### Leaching tests

Glass chemical durability is a direct measure of the wasteforms capability to immobilize its radionuclide content. The chemical durability depends on numerous factors such as composition, waste loading, leachate composition, pH rate, redox potential, diffusion coefficients, transport properties, the formation of surface layers, crystallization of the waste glass, phase separation and radiolysis^61,62^.

Concerning the stability of Ln-glasses, leaching tests in aqueous solutions were evaluated based on ASTM C1285-02 protocol^39,40^ on Matrix-Ln30 sample series. The ASTM’s PCT (Product Consistency Test) is generally used to investigate the chemical stability of nuclear glass waste form and study the release rate and kinetic characteristics of elements of interest. The glass samples were crushed in a ball mill and the tests were performed with the − 100 to + 200 mesh-size fraction. This static method is carried out in 304L stainless steel cylindrical containers at a constant temperature of 90 ± 1 °C using a laboratory oven. This temperature is higher than that expected to occur during the nuclear glasses alteration by groundwater (50 °C), enabling to increase leaching kinetic. In order to reach the saturation phenomena faster, the tests are generally performed with elevated surface to volume ratios. Following the ASTM protocol test A and test B were performed with a ratio of 10 ml/g regarding the leachate volume to the sample mass. The test A is conducted in strictly defined conditions, while test B allows applying different experimental parameters (temperature, duration, leachate volume to sample mass ratio, mesh size). During the test A after 7 days 6 ml, while during the test B after 3, 7 and 10 days 5 ml of aliquots were collected. The leachates were filtered through a 0,45 µm syringe filter and acidified with 20 µl of ultrapure cc. HNO_3_. The concentrations of the released elements were determined via ICP–OES (Inductively coupled plasma atomic emission spectroscopy). Normalized leaching rate was calculated with the following equation^39^:

4 $$NR_{i} = ~\frac{{c_{i} \left( {sample} \right)}}{{\left( {f_{i} } \right) \cdot \left( {\frac{{SA}}{V}} \right) \cdot \left( t \right)}}$$

where *c*_*i*_ is the concentration of the element in the given aliquot (g/l), *f*_*i*_ is the weight fraction of the element in the original glass form (-), $\frac{SA}{V}$ is the final waste form’s surface area divided by the volume of the leachate (m^-1^), *t* is the time duration (days).
